# Potential analgesic effect of Foshousan oil-loaded chitosan-alginate nanoparticles on the treatment of migraine

**DOI:** 10.3389/fphar.2023.1190920

**Published:** 2023-08-23

**Authors:** Yulong Chen, Qingzhou Cheng, Shan Zeng, Site Lv

**Affiliations:** ^1^ College of Medicine and Health Science, Wuhan Polytechnic University, Wuhan, China; ^2^ School of Mathematics and Computer Science, Wuhan Polytechnic University, Wuhan, China

**Keywords:** Foshousan oil, migraine, CS-AL NPs, inflammation, analgesia, vasoactive substances, hemorheology

## Abstract

**Background:** Migraine is a common neurovascular disorder with typical throbbing and unilateral headaches, causing a considerable healthcare burden on the global economy. This research aims to prepare chitosan-alginate (CS-AL) nanoparticles (NPs) containing Foshousan oil (FSSO) and investigate its potential therapeutic effects on the treatment of migraine.

**Methods:** FSSO-loaded CS-AL NPs were prepared by using the single emulsion solvent evaporation method. Lipopolysaccharide (LPS)-stimulated BV-2 cells and nitroglycerin (NTG)-induced migraine mice were further used to explore anti-migraine activities and potential mechanisms of this botanical drug.

**Results:** FSSO-loaded CS-AL NPs (212.1 ± 5.2 nm, 45.1 ± 6.2 mV) had a well-defined spherical shape with prolonged drug release and good storage within 4 weeks. FSSO and FSSO-loaded CS-AL NPs (5, 10, and 15 μg/mL) showed anti-inflammatory activities in LPS-treated BV-2 cells via reducing the levels of pro-inflammatory cytokines such as tumor necrosis factor-α (TNF-α), interleukin-1β (IL-1β), interleukin-6 (IL-6), and nitric oxide (NO), but elevating interleukin-10 (IL-10) expressions. Moreover, FSSO-loaded CS-AL NPs (52 and 104 mg/kg) raised pain thresholds against the hot stimulus and decreased acetic acid-induced writhing frequency and foot-licking duration in NTG-induced migraine mice. Compared with the model group, calcitonin gene-related peptide (CGRP) and NO levels were downregulated, but 5-hydroxytryptamine (5-HT) and endothelin (ET) levels were upregulated along with rebalanced ET/NO ratio, and vasomotor dysfunction was alleviated by promoting cerebral blood flow (CBF) in the FSSO-loaded CS-AL NPs (104 mg/kg) group.

**Conclusion:** FSSO-loaded CS-AL NPs could attenuate migraine via inhibiting neuroinflammation in LPS-stimulated BV-2 cells and regulating vasoactive substances in NTG-induced migraine mice. These findings suggest that the FSS formula may be exploited as new phytotherapy for treating migraine.

## 1 Introduction

Migraine is a chronic or episodic neurovascular disease characterized by a pulsatile intense headache in a unilateral or bilateral location with a variety of accompanying neurological symptoms including nausea, vomiting, photophobia, and phonophobia (Dodick, 2018). The epidemiological study shows that the global incidence of migraine was nearly 43% in women and 18% in men during the whole lifetime (Allais et al., 2018). Moreover, this neurovascular disorder with high morbidity and low recovery heavily debases the living quality of patients, which further results in huge direct costs with an estimated $11 billion in the healthcare budget of the United States (Ashina et al., 2021a). The trigeminal vascular hypothesis gradually becomes a compelling fundamental theory interpreting the pathogenesis of migraine, in which detrimental internal and external stimuli can cause vasomotor dysfunction and activate the trigeminal nervous system to release vasoactive substances and neurotransmitters, such as a strong vasodilator CGRP, and inflammatory mediators including IL-6 and TNF-α (Wattiez et al., 2020). Additionally, neurogenic inflammation is closely related to migraine attacks, and these pathophysiological alterations in turn underpin headache symptoms. It is noteworthy that vascular constriction may be associated with the upregulation of neurotransmitters like 5-HT, which contribute to the amelioration of neurogenic inflammation (de Vries et al., 2020). However, the exact mechanism is not yet clearly elucidated. Currently, clinical medication for migraine treatment has attracted spreading attention, in which triptans as serotonin receptor agonists have been developed as first-line therapy for patients suffering from migraine (Negro et al., 2018; Ferrari et al., 2022). Useful non-specific treatments such as non-steroidal anti-inflammatory drugs like aspirin and ergotamine are frequently accompanied by cardiovascular side effects and analgesic agents like opioids are strictly restrained due to drug abuse and addiction (Ashina et al., 2021b). In an FDA report, one new anti-migraine drug, aimovig, as a human monoclonal antibody antagonizing CGRP receptor, was assessed and approved in 2018 (Markham, 2018).

Foshousan is a famous traditional Chinese medicine (TCM) prescription widely used in the treatment of migraine and dysmenorrhea (Huang et al., 2013; Jin et al., 2016). It first appeared in Pu Ji Ben Shi Fang, a well-known herbal formula-recorded book written by Shuhui Xu during Song Dynasty. According to TCM theory, it is believed that headache is usually caused by wind cold, blood stasis, and nourishment deficiency (Hou et al., 2017). The Foshousan prescription is mainly composed of two single botanical drugs, namely, *Angelica sinensis* (Oliv.) Diels (Apiaceae; Angelica sinensis radix) and *Ligusticum chuanxiong* Hort. (Apiaceae; Chuanxiong rhizoma) in the mass ratio of 2:1. These two constituents are well-known to dispel wind, activate blood, and alleviate pain in TCM theory (Huang et al., 2020). Pharmacological studies reported that *Chuanxiong Rhizoma* with features of warmth in properties and pungent in flavor is capable of improving the circulation of blood and qi, expelling wind and relieving pain, especially headache and rheumatic arthralgia (Li et al., 2021). *Radix Angelica Sinensis* as another botanical drug was extensively used in the treatment for blood nourishing and dysmenorrhea (Jin et al., 2016; Li et al., 2023). A comparative analysis in the FSS formula revealed main aromatic acids (0.975 mg/g) and phthalides (5.178 mg/g) such as ferulic acid, butylidenephthalide, butylphthalide, ligustilide, senkyunolide A, senkyunolide H, and senkyunolide I (Li et al., 2014). Among them, ligustilide acting as one of the main bioactive compounds in the FSS formula accounted for 2.351 mg/g in content, and it was reported to exert anti-inflammatory and neuroprotective activities (Wu et al., 2020; Mao et al., 2022; Wu et al., 2022). The essential oil originating from *Radix Angelica Sinensis* showed analgesic effects and blood circulation improvements by adjusting glycine and arachidonic acid levels (Wang et al., 2016; Li et al., 2017; Hua et al., 2019). In addition, the volatile oil derived from *Chuanxiong Rhizoma* also exhibited anti-migraine effects by relieving pain behaviors (Guo-Jing et al., 2020; Yuan et al., 2021). Nevertheless, many active substances in essential oils with poor water solubility and high volatilization are prone to be unstable because of large surface areas of oil droplets available for oxidation, which may be avoided by nanoparticle encapsulation (Hou et al., 2022). Oil/Water emulsion is, therefore, utilized as a feasible strategy designed to suppress possible interactions between oil and oxygen to improve physical stability and pharmacological properties.

In recent decades, the application of micro-capsulation and nanoparticles as a drug delivery system has garnered numerous interests in the pharmaceutical field. Bio-based materials such as biocompatible and biodegradable polymers play a critical role in the formation of desirable nanoscale carriers that not only promote the accurate local targeting of drug release for a prolonged time but also achieve the development of green ecology (Baranwal et al., 2018; Tan et al., 2021). Alginate is a negatively charged, hydrophilic, natural polysaccharide composed of D-mannuronic acid and L-guluronic acid, and it produces a viscous gum when hydrated (Severino et al., 2019). Chitosan is a positively charged, linear polysaccharide consisting of D-glucosamine and N-acetyl-D-glucosamine, which is generated by the deacetylation of chitin under alkaline conditions. It is easily present in acidic solutions and also exerts good permeability in the transportation of hydrophilic substances (Rashki et al., 2021). In this research, a single emulsion solvent evaporation method was used to prepare CS-AL NPs containing FSSO. In the nano-emulsion, AL was one type of hydrophilic polymer functioning as a wall-forming material for protecting loaded volatile substances; moreover, CS and calcium chloride (CaCl_2_) are crosslinked with AL to produce hydrogel for gelification (Li S. et al., 2022).

**FIGURE 1:**
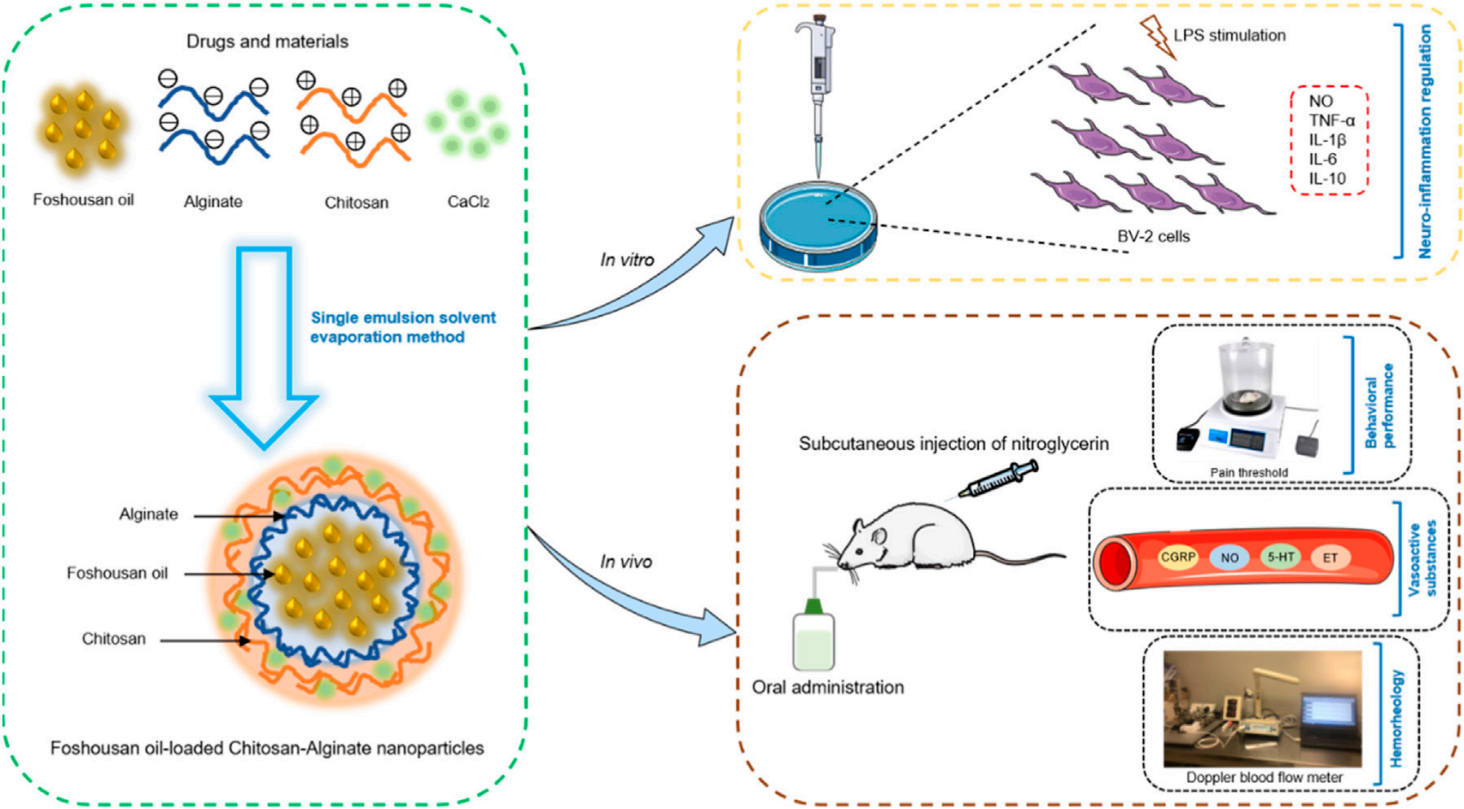
Schematic illustration of the preparation of FSSO-loaded CS-AL NPs and the investigation of their therapeutic actions in LPS-stimulated BV-2 cells and NTG-induced migraine mice.

In the study, FSSO nanoencapsulation was conducted through a single emulsion solvent evaporation method, which utilizes CS and AL as wall-forming biomaterials for enhancing physical stability. The feasibility of CS-AL NPs serving as important carriers for the delivery of FSSO was evaluated by determining key parameters including particle size (PS), polydispersity index (PDI), zeta potential, entrapment efficiency (EE), *in vitro* release rate, stability, and morphology. The optimal system during the preparation process was explored by varying the molecular weight (MW) of CS, the mass ratio of CS/AL, and the addition order of CS. Furthermore, as shown in Figure 1, this work aimed to investigate the anti-inflammatory and anti-migraine activities of FSSO-loaded CS-AL NPs in LPS-stimulated BV-2 cells and NTG-induced migraine mice, respectively.

## 2 Materials and methods

### 2.1 Materials

Alginic acid sodium salt from brown algae with MW of 80,000–120,000 Da, medium viscosity (≥2,000 cps) and low guluronic acid content (FG = 0.39) were purchased from Sigma (St. Louis, MO, United States). Chitosan with MW of 50,000 and 100,000 Da and a degree of deacetylation (≥90%) was supplied from Golden Shell Pharmaceutical Co., Ltd., (Zhejiang, China). Slices of *Radix Angelica Sinensis* and *Chuanxiong Rhizoma* were purchased from KangMei Pharmaceutical Company (Guangdong, China). The essential oil was isolated from the FSS formula composed of *Radix Angelica Sinensis* and *Chuanxiong Rhizoma* at a mass ratio of 2:1 by the supercritical fluid extraction technique. Thiazolyl blue tetrazolium bromide was purchased from Sigma (St. Louis, MO, United States). Mouse BV-2 microglial cells were obtained from Kunming Cell Bank, China Infrastructure of Cell Line Resources (Kunming, China). Detection kits used to determine IL-1β, IL-1, IL-10, TNF-α, NO, CGRP, 5-HT, and ET were purchased from Nanjing Jiancheng Bioengineering Institute (Nanjing, China). DiO was purchased from Beyotime Biotechnology Company (Shanghai, China). Sumatriptan (SUMA) was purchased from TRC Company (Toronto, Canada). Nitroglycerin was purchased from Hongrun Pharmaceutical Company (Henan, China). All other chemicals such as CaCl_2_, Tween-80, acetic acid, ethanol, and formalin were of analytical grade and procured from Aladdin (Shanghai, China). Methanol was of HPLC grade. Deionized distilled water was obtained from a Milli-Q system (Bedford, MA, United States).

### 2.2 Cell culture

The cells were cultured in Dulbecco’s Modified Eagle’s Medium (DMEM) supplemented with 10% (v/v) heated inactivated fetal bovine serum (FBS) and 1% (v/v) penicillin-streptomycin solution. The cells were incubated in a humid atmosphere containing 95% air and 5% CO_2_ at 37°C. The cells were passaged before confluence and the medium was replaced every 2 days (Kucic et al., 2019).

### 2.3 Animals

The adult female C57BL/6 mice (18–22 g) used in the study were obtained from the Laboratory Animal Center, University of Macau (Macau, China). All mice were housed in animal cages at a constant temperature (24°C ± 2°C) on a 12 h light/dark cycle with food and water available. All animal welfare and experiments were strictly performed in accordance with the Guide for the procedures approved by the Animal Ethics Committee, Institute of Chinese Medical Sciences, University of Macau (Approval No. UMARE-012M-2018).

### 2.4 Preparation of FSSO-loaded CS-AL NPs

NPs were prepared using the methods previously reported with modifications (Lertsutthiwong et al., 2009; Natrajan et al., 2015). There were three important steps in the single emulsion solvent evaporation method, including oil/water emulsification, gelification, and solvent removal. In brief, sodium alginate solution was prepared by dissolution in ultrapure water and the pH remained at the range of 5–5.5 to gain nanosized particles. The oil/water emulsion was conducted by adding dropwise 0.6 mL of volatile oil ethanolic solution (20 mg/mL) into 20 mL of AL solution (0.3 mg/mL) containing 1% (w/v) Tween-80, with subsequent sonication for 15 min. A measurement of 4 mL of 0.67 mg/mL CaCl_2_ solution was added dropwise to the mixture and continuously stirred for 30 min. After that, the emulsion was subsequently combined with 4 mL of 0.3 mg/mL CS solution containing 1% (v/v) acetic acid and stirred for additional 30 min. The pH of the CS solution was maintained between 4.5 and 5. The resulting suspension was then equilibrated overnight, and the solvent was removed by rotary evaporation at 40°C for 20 min. Eventually, FSSO-loaded CS-AL NPs were obtained as a dispersion in the aqueous solution.

### 2.5 Optimization and characterization of FSSO-loaded CS-AL NPs

The key factors implicated in the physical properties, such as the MW of CS (50,000 and 100,000 Da), the mass ratio of CS/AL (0:1, 0.1:1, 0.2:1, and 0.4:1), and the CS addition order (before or after CaCl_2_), were investigated for optimal formulation. The PS, PDI, and zeta potential were determined using a Malvern Zetasizer (Malvern Instruments Ltd., United Kingdom). The PS measurement based on the principle of dynamic light scattering showed its distribution in the range of 200–300 nm, with the scattering angle fixed at 90° and temperature controlled at 25°C. The zeta potential was detected through the laser doppler electrophoresis method. The EE and loading capacity (LC) were calculated through HPLC quantification analysis. After ultracentrifugation of NPs suspension at 14,800 rpm and 4°C for 30 min, the supernatant was collected and stored at 4°C for further analysis. The amount of FSSO encapsulated into NPs was assayed utilizing the HPLC method according to the study of Lertsutthiwong (Lertsutthiwong et al., 2009). In brief, the supernatant was diluted with methanol and injected into an Agilent extend-C18 column (4.6 mm × 250 mm, 5 μm) maintained at 25°C. The mobile phase was composed of methanol and ultrapure water (70:30). The injection volume was 10 μL. The isocratic elution was at a flow rate of 1.0 mL/min and detection was performed at a wavelength of 320 nm. The total time of chromatographic analysis was 23 min for each sample, with ligustilide eluting at a retention time of 12 min. A standard curve was plotted to determine the concentration of unknown oil samples. The entrapment efficiency and loading capacity were calculated using the following equations:

$$Entrapment\;efficiency\;\left(\%\right)=\frac{Total\;amount\;of\;oil-Free\;oil}{Total\;amount\;of\;oil}\times 100\%$$ (1)

$$Loading\;capacity\;\left(\%\right)=\frac{Total\;amount\;of\;oil-Free\;oil}{Weight\;of\;nanoparticle}\times 100\%$$ (2)

The *in vitro* drug release profiles were obtained by the equilibrium dialysis method. At first, the dialysis membrane bag was pre-soaked in deionized water for 1 h to remove the preservatives and then rinsed with phosphate-buffered saline (PBS) solution. NPs were re-dispersed in 2 mL of PBS solution at pH 7.4 and then placed in the dialysis bag. The dialysis membrane bag was sealed from both ends with clips and immersed in 50 mL of PBS containing 20% ethanol. The addition of ethanol can reduce the aggregation of oil droplets and facilitate their uniform release during the process (Xi et al., 2019). The time-dependent release study was carried out at time intervals of 0, 0.5, 1, 2, 4, 6, 8, 10, 12, 14, 16, 18, 20, 22, and 24 h. All sets were incubated at 37°C ± 0.5°C under gentle agitation at a speed of 50 rpm. At each time interval, an aliquot (2 mL) was withdrawn and quantified by HPLC analysis. The equivalent volume of fresh dissolution medium was then supplemented. The trial was repeated in triplicate. The release rate was calculated as follows:

$$Release\;\left(\%\right)=\frac{Released\;oil}{Total\;oil}\times 100\%$$ (3)

The assessment of physical stability was performed by measuring parameters including PS, PDI, zeta potential, and EE. The NPs suspension was stored at 4°C and 25°C for 4 weeks. These parameters were determined per week. The morphology was visualized using transmission electron microscopy (TEM, model JEM-1200EX, JEOL Ltd., Japan). The nanoscale particles were diluted with an appropriate volume of ultrapure water and might be sonicated to avoid aggregation. The sample was then placed on a Formvar-coated copper grid and negatively stained to minimize the background effect. The image from the copper grid was thereafter observed using high-resolution mode and maintained at 120 kV to confirm the shape and size.

### 2.6 Cytotoxicity assay

The toxicity in BV-2 cells was evaluated using the 3-(4,5-dimethylthiazol-2-yl)-2,5-diphenyl tetrazolium bromide (MTT) colorimetry method. This assay is commonly applied to determine the cell viability affected by the drug, which is based on the ability of living cells to produce formazan crystals on the substrate of MTT. The trial was performed according to the method provided by Bhunchu (Bhunchu et al., 2016). Briefly, the cells were seeded in a 96-well plate at a density of 1 × 10^4^ cells/well and incubated for 24 h before treatment. The cells were then exposed to FSSO-loaded CS-AL NPs at a variety of concentrations. Bare NPs were used to determine the cytotoxicity of biomaterials like CS and AL. The MTT reagent (1 mg/mL) was added to all the wells and incubated for 24 h. After careful removal of the MTT solution, dimethyl sulfoxide (DMSO) was then added to dissolve blue formazan particles and the plate was gently mixed for 10 min. A microplate reader (FlexStation 3, Molecular Devices, United States) was used to measure the absorbance at 570 nm. The cell viability was calculated with the following equation:

$$Cell\;viability\;\left(\%\right)=\frac{Nt}{Nc}\times 100\%$$ (4)

Where Nt represents the absorbance of cells treated with FSSO-loaded CS-AL NPs and Nc represents the absorbance of untreated cells.

### 2.7 Cellular uptake and intracellular distribution

To determine the intracellular localization, BV-2 cells were harvested with 1640 medium and seeded into a 20 mm glass bottom culture dish at a density of 3 × 10^5^ cells/mL/well. After the cells were incubated at 37°C for 12 h, the medium was removed. Free DiO and NPs containing DiO at predefined concentrations were then introduced into each well containing 1 mL of medium. After administration for 6 h, cells were treated with Hoechst for staining in the nucleus and incubated at room temperature for 30 min to avoid light. Cells were rinsed twice with PBS buffer at pH 7.4 and fixed with 4% paraformaldehyde for 15 min at the same condition as Hoechst dying. Eventually, cells were imaged by Leica TCS SP8 Confocal Laser Scanning Microscopy (Berlin, Germany). The cellular uptake of NPs was quantitatively measured by flow cytometry. BV-2 cells in 24-well plates were treated with free DiO and NPs containing DiO for 1, 3, and 6 h. The medium was then removed, and cells were washed with PBS and trypsinized. The cells were collected and centrifuged at 1,000 rpm for 10 min, washed with PBS twice, and resuspended in 1 mL of PBS. Then, cell suspensions were filtered and finally subjected to flow cytometry equipped with an argon laser at 488 nm and analyzed with software through the fluorescence channel.

### 2.8 Measurement of inflammatory factors levels

The effects of FSSO-loaded CS-AL NPs on LPS-induced neuroinflammation in BV-2 cells were assessed by the enzyme-linked immunosorbent assay (ELISA) method. In brief, cells were seeded in a 24-well plate at a density of 8 × 10^4^ cells/well and incubated for 24 h. Cells were pretreated with various concentrations (1, 5, 10, and 15 μg/mL) of NPs for 1 h, and then stimulated with 500 ng/mL LPS for another 24 h with the same concentration of drugs. After 24 h of exposure, the supernatant was carefully collected and centrifuged at 2,000 rpm and 4°C for 5 min. The final supernatant was stored in a tube at −20°C. The levels of TNF-α, IL-1β, IL-6, and IL-10 were determined by the commercial ELISA kits. The content of NO was measured by the detection kits using the colorimetric method.

### 2.9 NTG-induced migraine mice and drug administration

Forty-five C57BL/6 female mice were randomly divided into five groups (*n* = 9). Mice in the control, model, and positive groups were orally administrated with saline solution for successive 7 days, whereas FSSO-loaded NPs groups received intragastric administration of FSSO-loaded NPs at 52 and 104 mg/kg for the same period. Sumatriptan at 0.6 mg/kg was intraperitoneally injected into mice in the positive groups on day 7 after final administration. A preliminary trial was conducted to select effective doses. The whole timeline of the animal experiment is shown in Figure 5A. Based on the previous methods (Casili et al., 2020), subcutaneous injection of NTG at a dose of 10 mg/kg contributed to hyperalgesia and migraine in mice. Thirty minutes after the last administration, mice in the model, positive, and FSSO-loaded NPs groups received a subcutaneous injection of NTG solution (10 mg/kg), while mice in the control group were injected with an equivalent volume of vehicle. The migraine model was established successfully 30 min after the NTG injection. Afterward, the behavior of all mice was observed for 3 h.

### 2.10 Behavioral research

The hot-plate test was performed as the method previously described (Guo et al., 2013). Each female mouse was placed on a hot plate (Nanjing Calvin Biotechnology Company Ltd., Nanjing, China) at a constant temperature of 55°C ± 0.5°C to observe the main behavioral responses such as licking their hind paw and jumping. The latent time in seconds before the occurrence of response was recorded as the pain threshold. In the selection of acceptable animal subjects, mice that had a pain response latency of less than 10 s or greater than 60 s were excluded. The mice were tested twice prior to the administration and the mean value was considered as the baseline of pain threshold. The cut-off time was set to 60 s to protect mice from damage. The pain threshold of each mouse was individually determined at 30, 60, 90, and 120 min after NTG injection. The elevation of the pain threshold shows the analgesic effects of the drug. The acetic acid-induced writhing test was conducted according to the method described by Nakamura (Jha and Mishra, 2022). The mice were intraperitoneally injected with 0.6% acetic acid (10 mL/kg) 30 min following NTG injection. Then, the number of abnormal writhing movements was recorded for 15 min after acetic acid injection. The typical writhing reflex manifested as expanding the hind limb, constricting the abdomen, and raising the croup. The reduction of acetic acid-stimulated abnormal writhing responses indicates the analgesic effects of the drug. The formalin test was carried out according to the method reported by Bulboaca et al. (2017). The mice received 5% formalin solution (1 mL/kg) injected hypodermically in the right hindlimb 30 min after NTG injection. The total time of foot-licking response was recorded for 5 min. The decrease in foot licking time suggests the analgesic effects of the drug.

### 2.11 Determination of CGRP, 5-HT, ET, and NO levels

Four hours after model establishment, all animals were anesthetized with 10% chloral hydrate by intraperitoneal injection at the dosage of 3.5 mL/kg. The blood sample was collected from the orbital venous plexus and stored in tubes pre-treated with heparin and coagulated at room temperature. The blood sample was centrifuged at 4,000 rpm and 4°C for 10 min, and the supernatant was harvested for further analysis. Following the manufacturer’s instructions, serum NO level was measured by colorimetric method, while plasma CGRP, 5-HT, and ET levels were determined by the radioimmunoassay method with detection kits.

### 2.12 Hemorheology study

Each group (*n* = 3) of C57BL/6 female mice was used in the hemorheology study. The mice were anesthetized with 10% chloral hydrate at 3.5 mL/kg and then were fixed using stereotaxic apparatus. A midline incision (approximately 1 cm) was made with scissors on the skull of mice subject, and a laser Doppler flowmeter (Moor Instruments Ltd., Wilmington, United Kingdom) was perpendicularly attached to the parietal bone surface at a position of 2 mm posterior and 2 mm lateral from the bregma to monitor CBF in the left middle cerebral artery region (Hedna et al., 2015). After the surgery, the baseline of mice brain CBF was confirmed for 30 min. NTG was then subcutaneously injected for the establishment of the migraine model. Afterward, hemorheology parameters such as CBF, blood cell concentrations, and blood cell speed were continuously recorded for 60 min. A heating pad was used to maintain the normal temperature of laboratory mice.

### 2.13 Statistical analysis

All assays were performed in triplicate and data were represented as mean ± standard deviation (SD). Statistical analysis was executed by one-way ANOVA using the software GraphPad Prism version 9.0 (GraphPad Software Inc., San Diego, California) with a *p*-value <0.05 considered to indicate statistical significance.

## 3 Results

### 3.1 Characterization of FSSO-loaded CS-AL NPs

**FIGURE 2:**
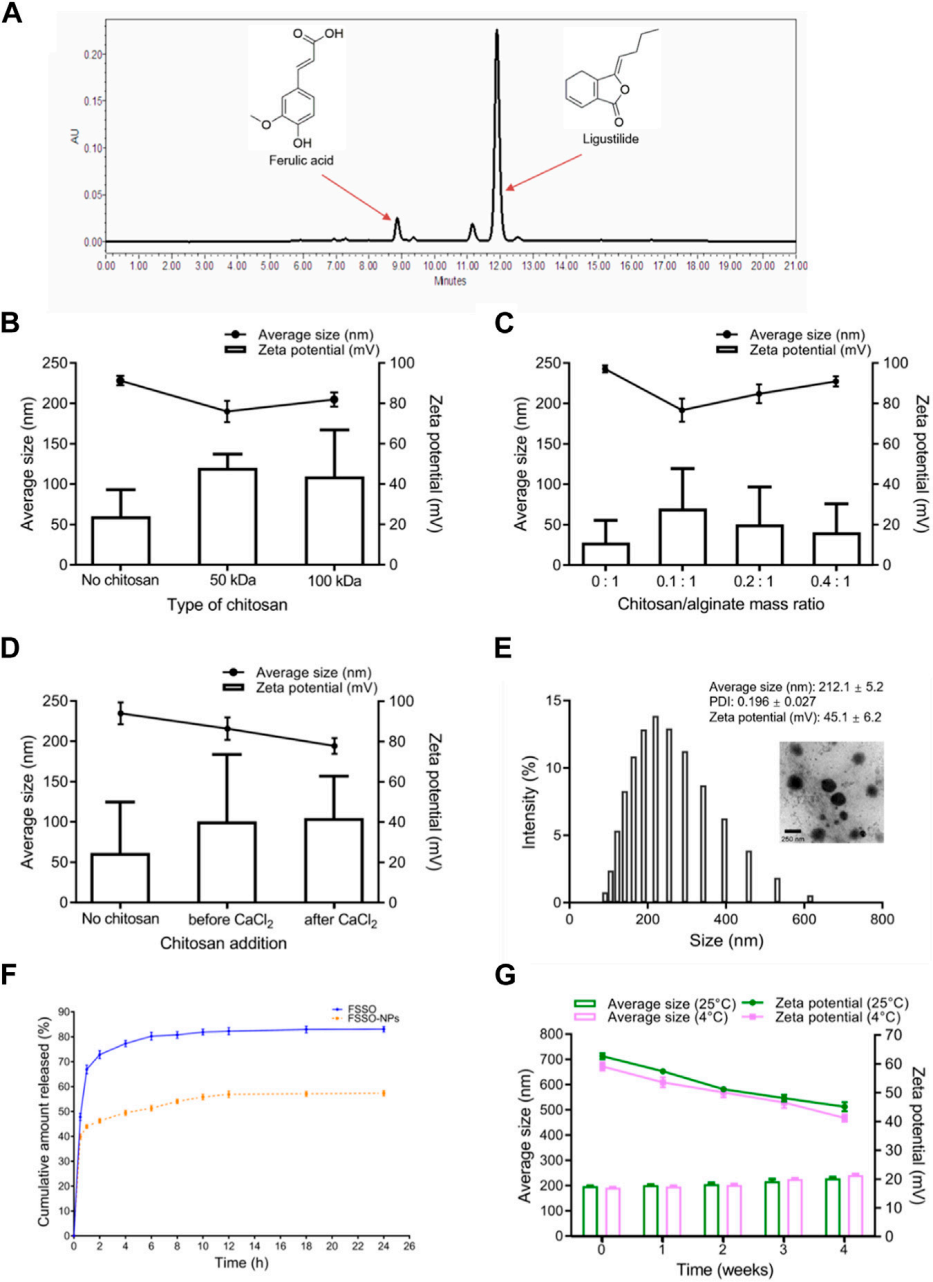
Characterization of FSSO-loaded CS-AL NPs. **(A)** HPLC chromatogram of ferulic acid and ligustilide in FSSO. **(B)** Effects of CS MW on FSSO-loaded NPs. **(C)** Effects of CS/AL mass ratio on FSSO-loaded NPs. **(D)** Effects of CS addition order on FSSO-loaded NPs. **(E)** Size distribution and morphology. **(F)** *In vitro* release study. **(G)** Stability study.

Effects of CS MW, CS/AL mass ratio, and CS addition order on average size and Zeta potential are shown in Figures 2B–D. In order to obtain smaller PS, optimal preparation conditions were dependent on CS with 50 kDa, CS/AL mass ratio of 0.1:1, and the addition of CS after CaCl_2_. Figure 2E indicates the size distribution of NPs with average size (212.1 ± 5.2 nm), PDI (0.196 ± 0.027), and Zeta potential (45.1 ± 6.2 mV) and also confirms that FSSO-loaded CS-AL NPs had a well-defined spherical shape with a diameter of approximately 220 nm. The results of *in vitro* release study are shown in Figure 2F. Compared with free FSSO released from ethanol solvent, FSSO-loaded NPs could prolong the time of drug release. As shown in Figure 2G, NPs experienced a slight elevation in PS and reduction in Zeta potential but still possessed good physical stability at 4°C and 25°C within 1 month.

### 3.2 Effects of FSSO-loaded CS-AL NPs on neurogenic inflammation in LPS-stimulated BV-2 cells

**FIGURE 3:**
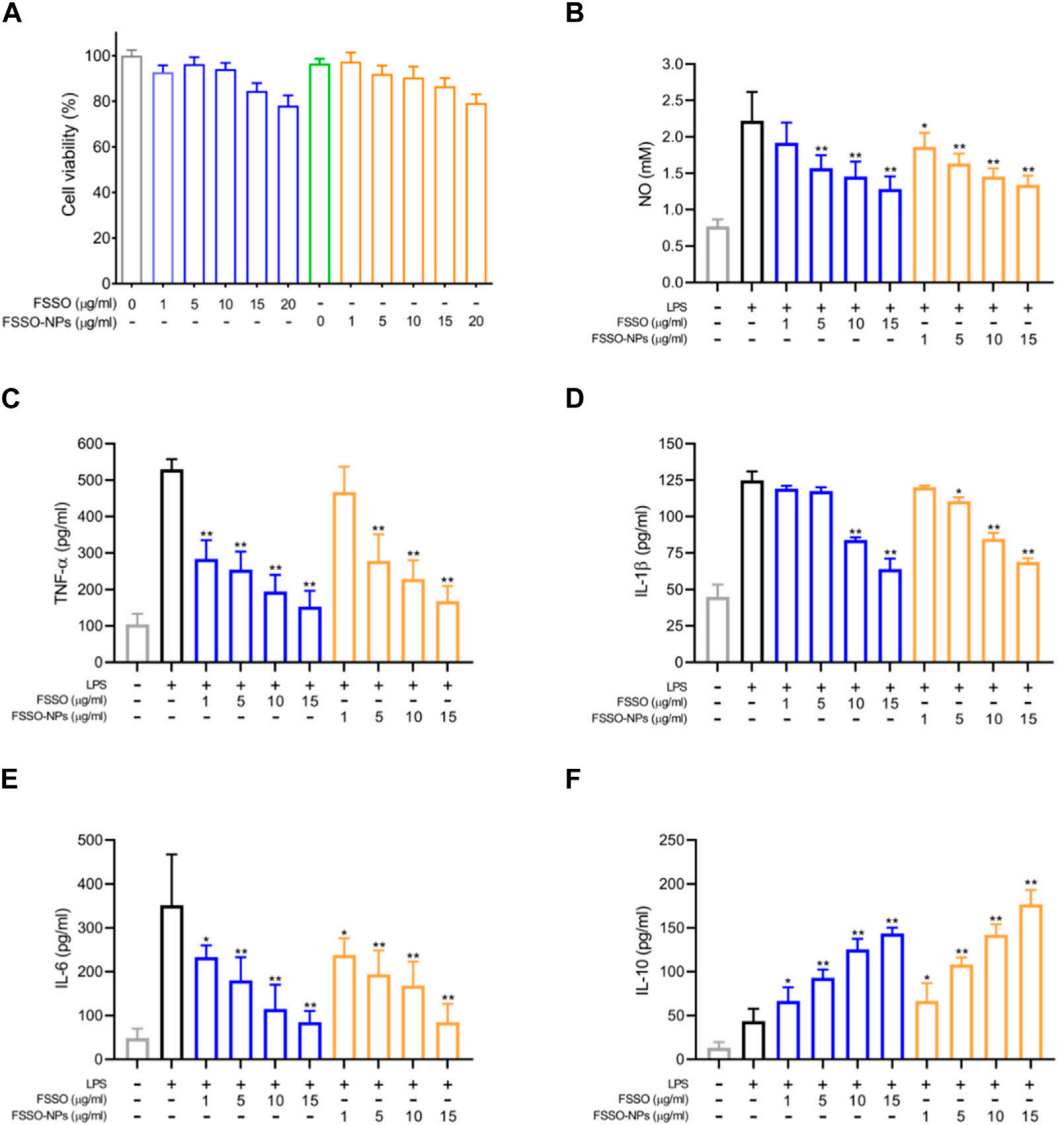
Effects of FSSO-loaded CS-AL NPs on neuroinflammation in LPS-induced BV-2 microglial cells. Cells were pretreated with various concentrations (1, 5, 10, and 15 μg/mL) of free FSSO and FSSO-loaded NPs for 1 h and then stimulated with 500 ng/mL LPS for another 24 h with the same concentration of drugs. The levels of TNF-α, IL-1β, IL-6, and IL-10 were measured by ELISA kits. The level of NO was determined by the detection kit. **(A)** MTT cell viability assay. **(B)** NO level. **(C)** TNF-α level. **(D)** IL-1β level. **(E)** IL-6 level. **(F)** IL-10 level. Data are presented as mean ± SD. (*n* = 3); *p < 0.05 and **p < 0.01 are significantly different compared with the LPS-treated group.

**FIGURE 4:**
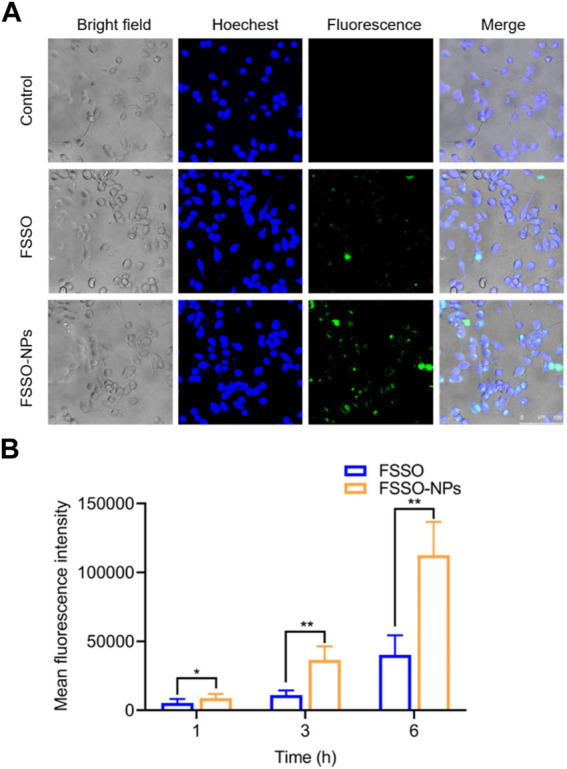
Cellular uptake and intracellular distribution of FSSO-loaded CS-AL NPs in BV-2 cells. **(A)** Intracellular distribution of free FSSO and FSSO-loaded NPs observed by One-photo Confocal Laser Scanning Microscopy. **(B)** Mean fluorescence intensity by Flow cytometry assays. Data are presented as mean ± SD. (*n* = 3); *p < 0.05 and **p < 0.01 are significantly different compared with the free FSSO group.

In Figure 3A, BV-2 cells treated with FSSO-loaded NPs (1, 5, 10, and 15 μg/mL) exhibited more than 90% cell viability, which suggested these dosages could be used in subsequent trials regarding inflammatory regulation. The levels of NO, TNF-α, IL-1β, and IL-6 were augmented significantly in BV-2 cells due to LPS stimulation. However, treatment with FSSO and FSSO-loaded NPs (1, 5, 10, and 15 μg/mL) inhibited the upregulated levels of pro-inflammatory mediators in a dose-dependent manner (Figures 3B–E). Besides, the same treatment also dose-dependently raised the levels of anti-inflammatory cytokine IL-10 (Figure 3F). As shown in Figure 4, FSSO-loaded NPs and free FSSO were absorbed into BV-2 cells. More importantly, the mean fluorescence intensity of the FSSO-loaded NPs group was much higher than that of the free FSSO group at 3 and 6 h.

### 3.3 Effects of FSSO-loaded CS-AL NPs on behavioral performance, vasoactive substances, and hemorheology in NTG-induced migraine mice

**FIGURE 5:**
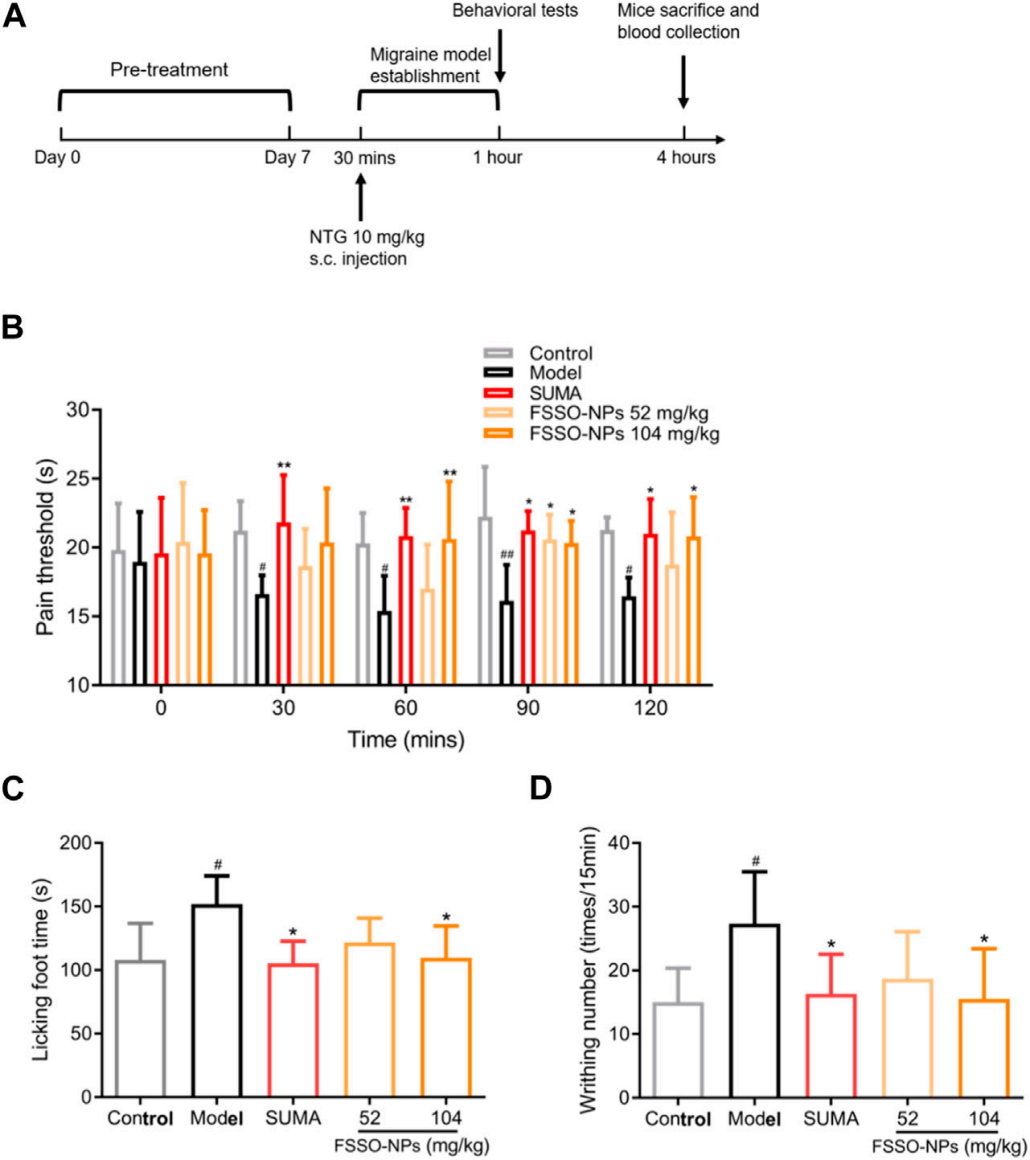
Effects of FSSO-loaded CS-AL NPs on behavioral performance in NTG-induced migraine mice. **(A)** Design and schedules of animal experiments. Each mouse was given FSSO-loaded NPs (52 and 104 mg/kg) or vehicle by intragastric route for successive 7 days. The migraine model was established by subcutaneous injection with NTG (10 mg/kg) 30 min after the last administration on day 7. Behavioral tests were conducted 30 min after NTG injection. All mice were anesthetized, and blood samples were collected 4 h after NTG injection. **(B)** Hot-plate test and heat-stimulated latent response time were recorded at 0, 30, 60, 90, and 120 min. **(C)** Formalin test and cumulative time of foot licking were recorded for 5 min after formalin injection. **(D)** The acetic acid-induced writhing test and the number of writhing responses were recorded for 15 min after 0.6% acetic acid injection. Data are presented as mean ± SD. (*n* = 6); ^#^ *p* < 0.05 and ^##^ *p* < 0.01 are significantly different compared with the control group. *p < 0.05 and **p < 0.01 are significantly different compared with the model group.

**FIGURE 6:**
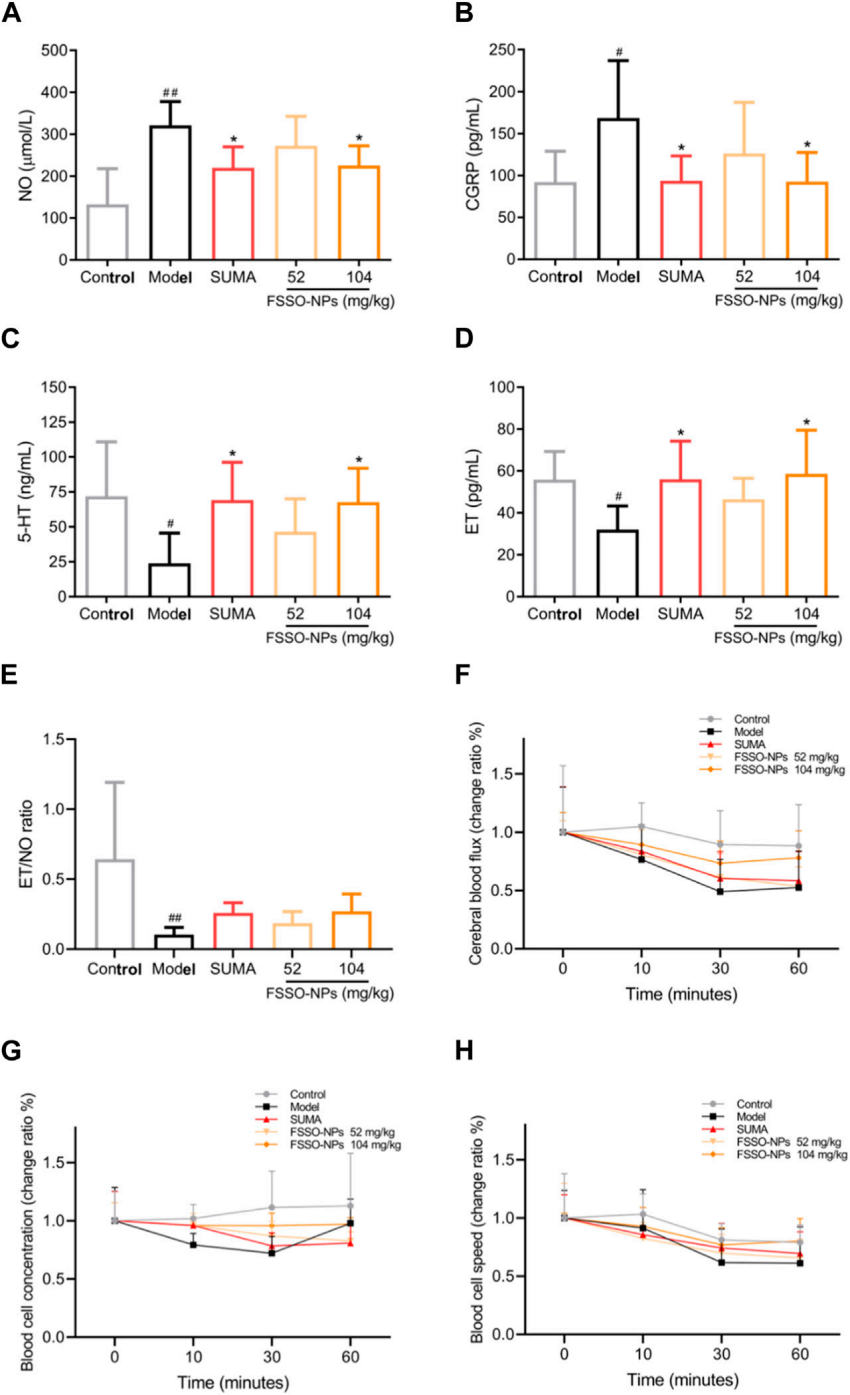
Effects of FSSO-loaded CS-AL NPs on vasoactive substances and hemorheology in NTG-induced migraine mice. **(A)** Serum NO level, **(B)** Plasma CGRP level, **(C)** Plasma 5-HT level, **(D)** Plasma ET level, and **(E)** ET/NO ratio. Data are presented as mean ± SD. (*n* = 6). **(F)** Change ratio of cerebral blood flux. **(G)** Change ratio of blood cell concentrations. **(H)** Change ratio of blood cell speed. Change rate (%) = 100% × (post-administration value—before administration value)/pre-administration value. Data are presented as mean ± SD. (*n* = 3). ^#^ *p* < 0.05 and ^##^ *p* < 0.01 are significantly different compared with the control group. *p < 0.05 and **p < 0.01 are significantly different compared with the model group.

The alterations of pain thresholds in the hot-plate test are shown in Figure 5B. NTG injection remarkably lowered pain thresholds when compared with the control group. FSSO-loaded NPs at 104 mg/kg could lengthen the hot-stimulus response latent time at 30, 90, and 120 min, while a low dose of 52 mg/kg administration increased pain thresholds only at 90 min. Sumatriptan at a dosage of 0.6 mg/kg significantly reversed NTG-caused decrement in response latency at all time points. In Figures 5C, D, administration of FSSO-loaded NPs at 104 mg/kg and sumatriptan decreased foot licking time and writhing response number compared with the model group. As shown in Figures 6A–E, the levels of serum NO and plasma CGRP in the model group were considerably elevated because of NTG induction. On the contrary, the levels of plasma 5-HT, ET, and the ET/NO ratio were decreased. Compared with the model group, the FSSO-loaded NPs at 104 mg/kg and sumatriptan administration significantly lowered the levels of serum NO and plasma CGRP and increased the levels of plasma 5-HT and ET, but the ET/NO ratio was not changed significantly. The alterations of CBF are shown in Figures 6F–H. The model group exerted a descending trend in CBF when compared with the control group. Nevertheless, only a high dose of FSSO-loaded NPs (104 mg/kg) rebalanced CBF. Similarly, blood cell concentration and speed in the model group were relatively lower than those of the control group, but the FSSO-loaded NPs group also restored the changes caused by NTG injection.

## 4 Discussion

The results indicated the therapeutical effects of FSSO in the treatment of migraine via various approaches. *Radix Angelica Sinensis* and *Chuanxiong Rhizoma* as major herbal ingredients of the FSS formula possessed blood nourishment, wind elimination, and pain alleviation in TCM perspectives (Huang et al., 2020). Furthermore, modern medical studies reported that these two Chinese medicines had anti-nociceptive and anti-inflammatory activities (Wang et al., 2020; Li C. et al., 2022). Tetramethylpyrazine (ligustrazine) from *Chuanxiong Rhizoma* showed an anti-inflammatory effect on cells by regulating related signaling pathways such as nuclear factor kappa-B (NF-κB) and PI3K/AKT (Zheng et al., 2013; Zhang et al., 2020; Huang et al., 2021). Ligustilide originated from both *Radix Angelica Sinensis* and *Chuanxiong Rhizoma* and also displayed analgesic and anti-inflammatory activities (Du et al., 2007; Wang et al., 2017; Song et al., 2023). In Figure 2A, ligustilide possessed a distinct peak at a retention time of approximately 12 min in the HPLC chromatogram of FSSO, indicating its dominant constituents in these two botanical drugs. Xie and colleagues made a comprehensive summary of the biological activities of ligustilide including anti-inflammation, anticancer, antioxidation, and neuroprotection (Xie et al., 2020). Nevertheless, potentially complicated correlations between bioactive ingredients and the pharmacological effects of FSSO are worth further investigation.

Several critical parameters involved in the preparation of FSSO-loaded CS-AL NPs affect their physical properties. For instance, CS with 50 kDa or a low concentration of CS solution can produce smaller NPs due to its low viscosity (Figures 2B, C). Li and colleagues reported that CS protonated amino groups that interacted with AL mannuronic-guluronic sequences often produced larger nanoparticles of randomly packed structure (Li C. et al., 2022). An increase in the average size of NPs with CS addition was an indication of the attachment of CS to AL on the surface of the FSSO core via electrostatic combinations, thereby generating a stable structure in the drug delivery system. However, NPs without CS addition had the largest PS, which implied that the intimate combination of CS and AL in the system may lead to particle compression and size reduction. As shown in Figure 2D, the addition of CS before CaCl_2_ forms smaller NPs, as evidenced by the fact that Ca^2+^ that strongly interacted with AL oligopolyguluronic sequences resulted in the formation of CaCl_2_-AL complex with compact egg-box structures (Lertsutthiwong et al., 2009; Xu et al., 2021). CS and AL solutions required pH values of 4.5-5 and 5-5.5, respectively. Hence, the influence of pH value on the CS-AL NPs containing FSSO needs further investigation. Based on the peak area of ligustilide in the FSSO chromatogram, the HPLC method was used to calculate the EE and LC of FSSO-loaded CS-AL NPs. Therefore, a standard curve (y = 5973620x – 206839, *R* ^2^ = 0.992) was obtained for calculating the oil content in unknown samples. The results suggested that optimal formulated NPs had high EE and LC with 86.9% ± 1.4% and 4.6% ± 0.1%, respectively. The utilization of CS in preparation provided higher EE and LC in comparison with those without CS, indicating that its addition could prevent drug leakage. Thus, a sustained release rate in NPs may be attributed to CS-induced shielding in the encapsulation of essential oil. Taken together, higher content of FSSO is captured into NPs due to complex membrane shells produced by biopolymers via electrostatic interactions between AL negatively charged carboxylic groups and CS positively charged amino groups. As these polyelectrolyte complexes stabilized the drug delivery system, FSSO-loaded CS-AL NPs maintained constant at 4°C and 25°C within 1 month (Figure 2G). In the cytotoxicity study, empty CS-AL NPs showed no toxicity in BV-2 cells, suggesting that these used polymers have good cytocompatibility as nanocarriers. Moreover, the cellular uptake study showed that the cellular fluorescence intensity of FSSO-loaded NPs was significantly higher than that of free FSSO. This may be attributed to CS-AL encapsulation that reaches into individual cells along with the transportation of botanical drugs. Therefore, CS-AL NPs are selected as suitable carriers for delivering FSSO and promoting potential bioactivities.

Inflammation is an immune response of neurological and vascular tissues against aggressive agents such as pathogens, irritants, and damaged cells (Conti et al., 2019; Erdener et al., 2021). It has been previously demonstrated that neuroinflammation in the central nervous system (CNS) contributed to the pathogenesis and progression of several neurogenic syndromes. Glial cells such as astrocytes, microglia, and oligodendrocytes play a pivotal role in the CNS based on their modulation of homeostasis (Ji et al., 2019). These cells are easily activated by diverse inflammatory stimuli, which in turn regulate the release of inflammation-associated cytokines like TNF-α, IL-1β, IL-6, and IL-10 and further cause neuronal impairment (Cobourne-Duval et al., 2018). Neuroinflammation evoked by activated microglia played a vital role in the pathophysiological alterations during interictal periods of migraine (Magni et al., 2019). Therefore, drugs that inactivate glial cells and suppress the production of proinflammatory factors might have therapeutic potential in the prevention and treatment of acute migraine. LPS stimulation uncovers that activation of the NF-κB signaling pathway is a critical step in inflammatory responses, which ultimately leads to iNOS expression and NO production (Yang et al., 2022). Furthermore, BV-2 mice microglial cells were treated with LPS to cause neuroinflammatory responses, which was often used to investigate anti-inflammatory activities of drugs in the management of migraine (Huang et al., 2018). As shown in Figures 3B–F, FSSO and FSSO-loaded NPs could dose-dependently downregulate the levels of NO, TNF-α, IL-1β, and IL-6 but heighten the levels of IL-10 in LPS-treated BV-2 cells, which indicated their potential protection against neuroinflammation. Therefore, it is inferred that FSSO-loaded NPs may have anti-inflammatory activities via modulating inflammation-related factors *in vitro*. However, whether their anti-inflammatory effects in LPS-treated BV-2 cells are related to the NF-κB signal pathway needs further investigations through determining the expressions of NF-κB p65, p-NF-κB p65, IκB, and p-IκB in Western blot analysis. The mRNA levels of inflammatory cytokines also require further measurement by qRT-PCR and immunofluorescence. Thus, these issues should be solved in a future work to explore better the anti-inflammatory mechanism of FSSO-loaded NPs in LPS-treated BV-2 cells.

Nociceptive sensitization is one of the typical symptoms in the occurrence of migraine (Luo et al., 2020). Thus, behavioral performance assessment was considered not only a critical observation index but also a successful signal of model establishment. In our study, three classical behavioral tests were used to evaluate the analgesic effects of FSSO-loaded NPs in NTG-induced migraine mice. The hot plate method as a popular behavioral evaluation is widely used in the detection of mice latent responses against acute thermal stimulus (Guo et al., 2013; Casili et al., 2020). As noxious hot stimulation could elicit supraspinal responses, this test usually reflected the central analgesic effects of drugs (Arslan et al., 2018). The results from the hot plate test revealed that administration of FSSO-loaded NPs at 104 mg/kg obviously inhibited the NTG-caused decline in pain thresholds, which was similar to Vong’s works (Vong et al., 2022). Interestingly, FSSO-loaded NPs at doses of 52 and 104 mg/kg exhibited sedative efficacy in migraine mice only at 90 min after NTG injection. Besides, sumatriptan as an effective anti-migraine drug prolonged the hot-stimulated response latent period at all times. Since visceral pain caused by acetic acid contributed to abnormal writhing in mice, an acetic acid-induced writhing test was used to verify the peripheral analgesic activities of drugs (Ferreira et al., 2013; Katole et al., 2022). Lower writhing frequency was observed after the administration of FSSO-loaded NPs at 104 mg/kg. Meanwhile, the formalin test was adopted to induce neuropathic pain in the animal experiment. Formalin-injected mice displayed nociceptive sensitive characteristics such as foot licking, limping, and jumping. The results showed that FSSO-loaded NPs at a high dose of 104 mg/kg significantly diminished the total time of foot licking in migraine mice. Based on the above results, FSSO-loaded NPs considerably improved behavioral abnormality via increasing pain thresholds, thereby highlighting their central and peripheral analgesic effects in NTG-induced migraine mice.

According to the most acknowledged trigeminal vascular theory, cerebral vascular dysfunction intervened by vasoactive substances and neurotransmitters such as CGRP, NO, 5-HT, and ET plays a pivotal role in the pathogenesis of migraine, which is frequently accompanied by neuroinflammation (Ramachandran, 2018; Yamanaka et al., 2021). The metabolic disorders of these vasoactive substances and neurotransmitters can contribute to the development of migraine. CGRP serving as a strong vasodilator neuropeptide is released when the trigeminal nerve is stimulated. This release further causes vasorelaxation, plasma protein extravasation, and mase cell degranulation, contributing to neurogenic inflammatory responses (Russo, 2015; Cauchi and Robertson, 2016; Gimeno-Ferrer et al., 2022). It is noteworthy that CGRP release can be suppressed by the 5-HT1B/D receptors agonist sumatriptan which has been applied in the clinical pharmacotherapy for migraine. CGRP also leads to nociceptive sensitization and thus aggravates pain symptoms like headaches during migraine attacks (Khan et al., 2019). In clinical studies, migraine attacks are frequently accompanied by a significant ascending in the levels of plasma CGRP (Wrobel Goldberg and Silberstein, 2015; Bhakta et al., 2021; Russo and Hay, 2023). Furthermore, due to the activation of TRPA1 and TRPV1 receptor channels, the synthesis and release of CGRP from trigeminal neurons are promoted by another vasoactive agent NO that primarily regulates CBF and arterial diameters. In turn, CGRP is able to stimulate primary cultures of trigeminal neurons to produce NO and iNOS (Capuano et al., 2014; Iyengar et al., 2019). NTG as NO-donor produces endogenous NO in animals, which further causes the relaxation of smooth muscle and the dilation of cerebral blood vessels via activating the soluble guanylate cyclase (Beuve et al., 2016; Pradhan et al., 2018). NO also participates in the mediation of neurogenic inflammation that might precipitate the progression of migraine (Ramachandran, 2018). In addition, the enhanced production of 5-HT and ET as important neurotransmitters in the management of migraine potentiates the inhibitory modulation of nociceptive signaling transmission (Vila-Pueyo et al., 2022; Vong et al., 2022). The increased levels of 5-HT contribute to vasoconstriction in the prodromal phase of migraine, while reduced content of 5-HT cannot sustain vascular constriction and further causes hemangiectasis in the ictal stage of migraine. The 5-HT downregulation also leads to a descent in pain thresholds within the thalmencephalon (Wang et al., 2011; Liu et al., 2020). Based on the above views, vasoactive substances and neurotransmitters were determined to investigate the potential effect of FSSO-loaded NPs in NTG-induced migraine mice. This study demonstrated that FSSO-loaded NPs at 104 mg/kg remarkably downregulated the levels of plasma CGRP and serum NO, and raised the ET/NO ratio in migraine mice. Moreover, the levels of plasma 5-HT and ET in the FSSO-loaded NPs-treated group were higher than those in the model group. Interestingly, there was a negative relationship between the contents of plasma CGRP and ET. The ET/NO ratio displayed a recovered trend in NTG-induced migraine mice after FSSO-loaded NPs administration. These findings implied that the anti-migraine effects of FSSO-loaded NPs may be intimately associated with the adjustment of vasoactive substances and neurotransmitters in the plasma of NTG-injected mice, which is conducive to attenuating potential neuroinflammation. Moreover, cerebral hemorheology tended to be abnormal in the development of migraine (Brennan and Charles, 2010; Knol et al., 2020). Vasomotor dysfunction stimulated the trigeminal neurovascular system to release more vasoactive substances such as CGRP and NO, which in turn resulted in cerebrovascular disturbances and neuroinflammation. In the study, the laser doppler flowmetry technique was selected to measure the changes in CBF in migraine mice. The results demonstrated that FSSO-loaded NPs at a high dose of 104 mg/kg significantly enhanced CBF, blood cell concentration, and speed. Their therapeutic benefits on migraine may be linked to the improvement of vasomotor dysfunction. Combined with the modulation of vasoactive substances and neuropeptides reported in experimental data, the trigeminal vascular theory was therefore strongly supported in the pathophysiology of migraine. However, several points need to be further investigated in terms of current study results. Since c-fos functions as an important indicator of neuronal activation, its expression reflects direct neuronal hyperexcitability and further neurological correlations during nociceptive transmission (Kilinc et al., 2015; Miyamoto et al., 2019; Brown et al., 2022). Hence, the overexpression of c-fos in brain tissues such as the brainstem, hypothalamus, and trigeminal nucleus caudalis should be investigated by the immunohistochemistry method. It is necessary to further explore whether the anti-nociceptive channel in the periaqueductal gray is blocked and whether some related mediators such as enkephalin, opioid receptors, and cholecystokinin are regulated. Whether behavioral symptoms like anxiety and photophobia in migraine animal models are relieved after drug administration also needs to be validated. Moreover, the role played by the antioxidant system in the pathophysiology of migraine deserves more studies in which Nrf-2, HO-1, and COX-2 expressions in the cerebrum should be observed by Western blot analysis. Hence, these points provide valuable directions for future research.

## 5 Conclusion

The present study revealed the therapeutic effect of FSSO on migraine and investigated the underlying mechanisms. We demonstrated that FSSO-loaded CS-AL NPs showed anti-inflammatory effects in LPS-induced BV-2 cells by regulating the levels of inflammatory cytokines such as TNF-a and IL-1β. In addition, their oral administration could alleviate the pain response in NTG-induced migraine mice, and migraine-related vasoactive substances and neurotransmitters such as CGRP and 5-HT were significantly altered in the FSSO-loaded CS-AL NPs high-dose group. The hemorheology study implied that anti-migraine treatment of FSSO-loaded CS-AL NPs was highly associated with promoted CBF. Taken together, these findings suggested that FSSO-loaded CS-AL NPs exhibit potential analgesic effects in migraine mice through regulating vasoactive substances and improving cerebral hemorheology.

## Figures and Tables

**Table:** 

**CaCl**_**2**_	Calcium chloride
**CBF**	Cerebral blood flow
**CGRP**	Calcitonin gene-related peptide
**CNS**	Central nervous system
**CS-AL**	Chitosan-Alginate
**DMEM**	Dulbecco’s Modified Eagle’s Medium
**DMSO**	Dimethyl sulfoxide
**EE**	Entrapment efficiency
**ELISA**	Enzyme-linked immunosorbent assay
**ET**	Endothelin
**FBS**	Fetal bovine serum
**FSSO**	Foshousan oil
**5-HT**	5-hydroxytryptamine
**IL-1β**	Interleukin-1β
**IL-6**	Interleukin-6
**IL-10**	Interleukin-10
**LC**	Loading capacity
**LPS**	Lipopolysaccharide
**MTT**	3-(4,5-dimethylthiazol-2-yl)-2,5-diphenyl tetrazolium bromide
**MW**	Molecular weight
**NF-κB**	Nuclear factor kappa-B
**NO**	Nitric oxide
**NPs**	Nanoparticles
**NTG**	Nitroglycerin
**PBS**	Phosphate-buffered saline
**PDI**	Polydispersity index
**PS**	Particle size
**SD**	Standard deviation
**TCM**	Traditional Chinese medicine
**TEM**	Transmission electron microscopy
**TNF-α**	Tumor necrosis factor-α

## Data Availability

The original contributions presented in the study are included in the article/Supplementary Material, further inquiries can be directed to the corresponding author.
